# The Expression of Fibroblast Activation Protein in Clear Cell Renal Cell Carcinomas Is Associated with Synchronous Lymph Node Metastases

**DOI:** 10.1371/journal.pone.0169105

**Published:** 2016-12-29

**Authors:** Peio Errarte, Rosa Guarch, Rafael Pulido, Lorena Blanco, Caroline E. Nunes-Xavier, Maider Beitia, Javier Gil, Javier C. Angulo, José I. López, Gorka Larrinaga

**Affiliations:** 1 Department of Physiology, University of the Basque Country (UPV/EHU), Leioa, Bizkaia, Spain; 2 Department of Nursing I, School of Nursing, University of the Basque Country (UPV/EHU), Leioa, Bizkaia, Spain; 3 Cancer Biomarkers Group, BioCruces Health Research Institute, Barakaldo, Bizkaia, Spain; 4 Department of Pathology, Complejo Hospitalario B de Navarra, Pamplona, Navarra, Spain; 5 Ikerbasque, Basque Foundation for Science, Bilbao, Bizkaia, Spain; 6 Department of Tumor Biology, Institute for Cancer Research, Oslo University Hospital Radiumhospitalet, Oslo, Norway; 7 Department of Urology, Hospital de Getafe, Universidad Europea de Madrid, Madrid, Spain; 8 Department of Pathology, Cruces University Hospital, University of the Basque Country (UPV/EHU), Barakaldo, Bizkaia, Spain; Queen Mary Hospital, HONG KONG

## Abstract

Clear cell renal cell carcinoma (CCRCC) is a heterogeneous and complex disease that frequently develops distant metastases. Fibroblast activation protein (FAP) is a serine peptidase the expression of which in cancer-associated fibroblasts has been associated with higher risk of metastases and poor survival. The objective of this study was to evaluate the role of FAP in metastatic CCRCC (mCCRCC). A series of 59 mCCRCC retrospectively collected was included in the study. Metastases developed either synchronous (n = 14) or metachronous to renal disease (n = 45). Tumor specimens were obtained from both primary lesion (n = 59) and metastases (n = 54) and FAP expression was immunohistochemically analyzed. FAP expression in fibroblasts from primary tumors correlated with FAP expression in the corresponding metastatic lesions. Also, primary and metastatic FAP expression was correlated with large tumor diameter (>7cm), high grade (G3/4), high stage (pT3/4), tumor necrosis and sarcomatoid transformation. The expression of FAP in primary tumors and in their metastases was associated both with synchronous metastases and also with metastases to the lymph nodes. FAP expression in the primary tumor was correlated with worse 10-year overall survival. Immunohistochemical detection of FAP in the stromal tumor fibroblasts could be a biomarker of early lymph node metastatic status and therefore could account for the poor prognosis of FAP positive CCRCC.

## Introduction

Renal cancer ranks within the top-ten list of the most frequent malignancies in Western Countries. Epidemiological data reveal that its incidence has been increasing in Europe and United States during the last years [1,2]. Clear cell renal cell carcinoma (CCRCC) is the most common histological subtype, accounting approximately for 75–80% of the cases [3]. CCRCC is frequently resistant to current chemo- and radiotherapy, and the standard treatment is complete resection of the primary tumor by radical or partial nephrectomy [4].

At the time of diagnosis approximately 25% of the patients display locally advanced or metastatic disease and about 33% of organ-confined tumors will develop metastatic disease [5]. Although immunotherapy and other targeted therapies have recently provided promising results, patients with metastatic CCRCC (mCCRCC) still have a poor prognosis [6]. Therefore, biomarkers associated with metastatic status in CCRCC are important for early detection and for the identification of new therapeutic targets [6,7].

Cancer-associated fibroblasts (CAFs) are the most ubiquitous elements of tumor stroma and are found in numerous types of solid cancers [8]. The cross-talk between neoplastic cells and CAFs in tumor microenvironment is crucial in CCRCC progression, invasion and metastasis, and in the acquisition of drug resistance [9]. A hallmark of the activation of these CAFs is the cell surface expression of fibroblast activation protein-α (FAP), a serine peptidase with multifunctional properties [10]. The overexpression of FAP in CAFs and in tumor cells has been associated with higher risk of metastases and worse survival in several solid tumors [9,11].

A recent study has demonstrated that the interaction of renal cancer cell lines with CAFs stimulates proliferation, survival and migration of tumor cells, thus indicating that these stromal cells play an important role supporting and promoting renal cancer progression [12]. However, FAP expression in renal cell carcinomas has not been previously documented in the literature. Very recently, we described the expression of this protein in a series of CCRCC, and demonstrated a correlation between FAP immunostaining in CAFs and poor patient outcome [13].

For a better understanding of the prognostic implications of this serine peptidase in renal cancer, we analyzed the immunohistochemical expression of FAP in CAFs both in primary tumors and in their metastases from a series of CCRCC.

## Materials and Methods

The authors declare that all experiments carried out in this study comply with current Spanish and European Union legal regulations. Samples and data from patients included in this study were provided by the Basque Biobank for Research-OEHUN (www.biobancovasco.org). All patients were informed about the potential use for research of their surgically resected tissues, and accepted this eventuality by signing a specific document approved by the Ethical and Scientific Committees of the Basque Country Public Health System (Osakidetza) (CEIC 2015/060, CEIC-E PI2015101).

### Patients and tissue specimens

59 patients with mCCRCC were included in this retrospective study. Primary tumors and metastases originated from the same patients and were surgically excised in every case. Clinical and pathological data of the selected samples are summarized in Table 1. Males predominated in the series (45M/14F). Mean age was 59 years. Follow-up data was obtained from the clinical records and was closed at Dec 31, 2014. At that time, 38 patients (64%) had died of disease. Mean follow-up was 65 months. Average tumor diameter of primary tumors was 7.9 cm (range 2-19cm). Twenty-four cases were low-grade (G1-2) and 34 high-grade (G3-4). At the time of diagnosis 32 cases were organ-confined (pT1/2) and 27 locally advanced or metastatic (pT3/4). Sarcomatoid changes were detected in 4 cases and tumor necrosis in 30.

**Table 1:** Clinical and pathological parameters of CCRCC patients (n = 59).

Patients	Average (%)
**Age (range)**	59 (25–83)
**Sex**	
Male	45 (76%)
Female	14 (24%)
**Follow-up (months)**	65 (1–240)
**Survival**	
Alive	21 (36%)
Dead of disease	38 (64%)
**Primary tumor**	**n**
**Diameter**	
≤ 7cm	34 (58%)
> 7cm	25 (42%)
**Grouped grade**	
Low (G1-G2)	24 (41%)
High (G3-G4)	35 (59%)
Grouped pT*	
Low (pT1-pT2)	32 (54%)
High (pT3-pT4)	27 (46%)
**Sarcomatoid**	
No	55 (93%)
Yes	4 (7%)
**Necrosis**	
No	29 (49%)
Yes	30 (51%)
**Metastases**	**n**
Synchronous	14 (24%)
Metachronous	45 (76%)
**Location**	
Lymphatic nodes	12 (20%)
Epithelial tissues	31 (53%)
Soft/bone tissues	16 (27%)

(*) Grouped pT: organ confined (pT1-pT2) vs non organ confined (pT3-pT4) disease

With regard to the metastatic status, 14 cases displayed synchronous metastases at the time of diagnosis and 45 developed metachronous metastatic disease after renal surgery. Thirty-one cases metastasized in epithelial organs, 16 in the soft tissues and bones and 12 in lymph nodes. Primary tumors and metastasis were surgically excised in every case. However, metastatic tissue available for immunohistochemical analysis was limited to 54 cases since the obtained samples in five cases were very scarce (core biopsies) and exclusively allowed the diagnosis on hematoxylin-eosin sections. Cases were reviewed by two pathologists (JIL, RG), who assigned Fuhrman’s grade [14] and 2010 AJCC Staging System [15].

### Immunohistochemistry assay

Immunohistochemistry with carbonic anhydrase IX (Epitomics, ref. code AC-0137RUO, dilution 1: 100), CK7 (Ventana, ref. code 790–4462, ready to use) and CD117 (Ventana, ref. code 790–2951, ready to use) was performed to confirm the diagnosis of CCRCC only in selected cases. Tissue microarrays were performed for the evaluation of FAP expression in primary and metastatic tumors. The presence/absence of FAP (Rabbit polyclonal to Fibroblast Activation Protein alpha, Abcam, ref. ab53066, dilution 1:70) was evaluated in the stromal fibroblasts adjacent to neoplastic nests. Immunostainings were performed in automated immunostainers (EnVision FLEX, Dako Autostainer Plus; Dako, Glostrup, Denmark and BenchMark Ultra, Ventana Medical Systems, Tucson, AZ, USA) following routine methods. Tris-EDTA was used for antigen retrieval in all cases. Negative controls were slides not exposed to the primary antibody, and these were incubated in PBS and then processed under the same conditions as the test slides. The analysis was performed using a Nikon Eclipse 80i microscope (Tokyo, Japan).

### Western-blot analysis

1 μg of human recombinant FAP protein (CF 3715-SE-010, R&D Systems) was loaded in a SDS-PAGE gel and transferred onto a PVDF membrane. The band was marked with a rabbit antibody against FAP (ab53066, 1:500, Abcam). The band was then detected with HRP-conjugated goat anti-rabbit antibody (sc-2004; Santa Cruz Biotechnology; 1:1000) and visualized with ECL substrate (S1 Fig).

### Statistical analysis

SPSS^®^ 21.0 software was used for the statistical analysis. Chi-square (χ^2^) test was used to analyze the association between categorical FAP expression (negative or positive) and pathological variables of CCRCCs. Spearman rho test was used to correlate FAP expression in both primary tumors and metastases, and with patients age and sex. Kaplan-Meier curves and log-rank test were performed to evaluate 10-year overall survival of the series according to the status of FAP expression in the primary tumor and in the metastases. Multivariate analysis was used to test the independent effects of clinical and pathological variables on survival. A stepwise selection procedure (forward conditional method) with a threshold entry of p = 0.20 and a stay criterion of p = 0.20 was used to select the optimal predictive model.

## Results

### Immunohistochemical expression of FAP in tumors

**Fig 1:**
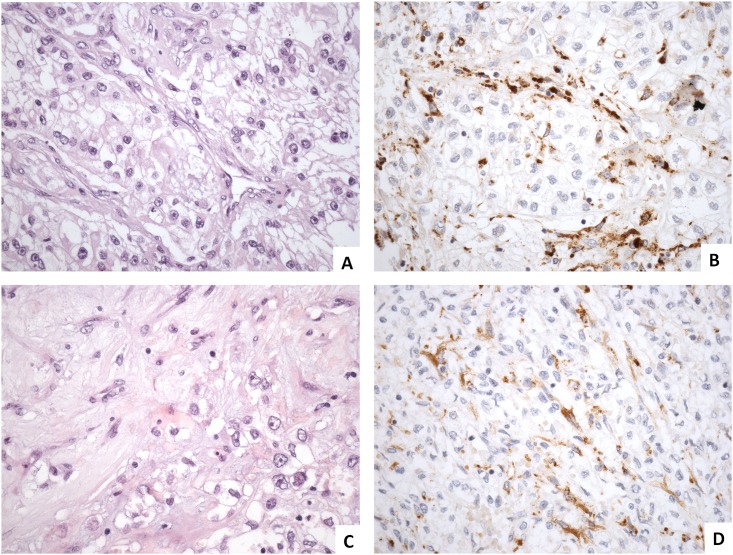
FAP immunohistochemical expression in primary and metastatic CCRCC. Clear cell renal cell carcinoma arranged in nests (A) show FAP-positive cancer-associated fibroblasts delineating tumor lobes (B). Lymph node metastatic tumor growing more diffusely without recognizable architecture (C) displays FAP-positive cancer-associated fibroblasts intimately intermingled with tumor cells (D).

Histologically, FAP expression was evaluated in the fibroblasts of the tumor stroma in both primary and metastatic tumors (Fig 1). The staining was cytoplasmic and involved exclusively the stromal fibroblasts adjacent to neoplastic epithelial cells, with a pattern of distribution that has been previously described [13].

### FAP expression according to clinical and pathological variables

FAP expression correlated positively in paired primary tumors and metastases (Spearman rho test r = 0.51; p = 0.0001). FAP was positive in 36% primary and 44% metastatic lesions. A statistically significant association was found between FAP expression and pathological parameters of tumor aggressiveness (Tables 2 and 3). Thus, FAP immunostaining was higher in high grade and high stage primary CCRCCs, and in tumors showing sarcomatoid transformation and necrosis (Table 2). FAP expression in metastases showed similar correlations with these variables (with the exception of tumor grade), but also correlated with higher tumor diameter (Table 3). On the other hand, FAP was not correlated with age and sex (Spearman rho test p>0.05).

**Table 2:** Clinical and pathological variables and FAP expression in primary tumors.

Variables	Negative (%)	Positive (%)	Total (n)	P value
**Diameter**				
≤ 7cm	74	26	34	0.088
> 7cm	52	48	25	
**Grade**				
Low (G1-G2)	88	12	24	**0.002**
High (G3-G4)	49	51	35	
**Grouped pT**				
Low (pT1-pT2)	81	19	32	**0.003**
High (pT3-pT4)	44	56	27	
**Sarcomatoid**				
No	69	31	55	**0.005**
Yes	0	100	4	
**Necrosis**				
No	86	14	29	**0.001**
Yes	43	57	30	
**Metastases**				
Synchronous	14	86	14	**0.0001**
Metachronous	80	20	45	
**Location**				
Lymph nodes	25	75	12	**0.005**^(a)^
Epithelial tissues	77	23	31	
Soft/bone tissues	69	31	16	

^**(a)**^ FAP expression was significantly higher in metastatic lesions from lymphatic nodes than from epithelial and soft/bone tissues.

Intergroup differences were evaluated using χ^2^ test. Statistically significant correlations are highlighted in bold.

**Table 3:** Clinical and pathological variables and FAP expression in metastases.

Variables	Negative (%)	Positive (%)	Total (n)	P value
**Diameter**				
≤ 7cm	70	30	30	**0.017**
> 7cm	37	73	24	
**Grade**				
Low (G1-G2)	65	35	23	0.22
High (G3-G4)	48	52	31	
**Grouped pT**				
Low (pT1-pT2)	72	28	29	**0.007**
High (pT3-pT4)	36	64	25	
**Sarcomatoid**				
No	59	41	51	**0.046**
Yes	0	100	3	
**Necrosis**				
No	78	22	27	**0.001**
Yes	33	67	27	
**Metastases**				
Synchronous	17	83	12	**0.002**
Metachronous	67	33	42	
**Location**				
Lymph nodes	31	69	13	**0.021**^(a)^
Epithelial tissues	69	31	29	
Soft/bone tissues	50	50	12	

^**(a)**^ FAP expression was significantly higher in metastatic lesions from lymphatic nodes than from epithelial tissues.

Intergroup differences were evaluated using χ^2^ test. Statistically significant correlations are highlighted in bold.

With regard to the temporal presentation of metastases, 14 were synchronous and 45 metachronous. In tumors with synchronous metastases FAP was expressed in 86% of the primaries and in 83% of the metastases (Table 2). By contrast, FAP was only positive in 20% of the primaries and in 33% of the metastases when they were metachronous (Table 3).

With regard to the metastatic location, 50% of the synchronous metastases were detected in the lymph nodes, 29% in epithelial organs and 21% in the soft tissues and bones. Conversely, metachronous metastases appeared mainly in epithelial organs (59%), followed by the soft tissues and bones (28%) and lymph nodes (13%). This different temporal topographic distribution was statistically significant (Chi-square χ^2^ test; p = 0.012).

Up to 75% of primary tumors that metastasized into lymph nodes were FAP positive. By contrast, primary tumors that metastasized into the soft tissues and bones and into epithelial organs were FAP positive only in 33% and 25%, respectively (Table 2). FAP expression in metastases showed a similar pattern, being significantly higher in lymph nodes than in epithelial organs (Table 3).

### FAP expression according to 10-year overall survival of mCCRCC patients

**Fig 2:**
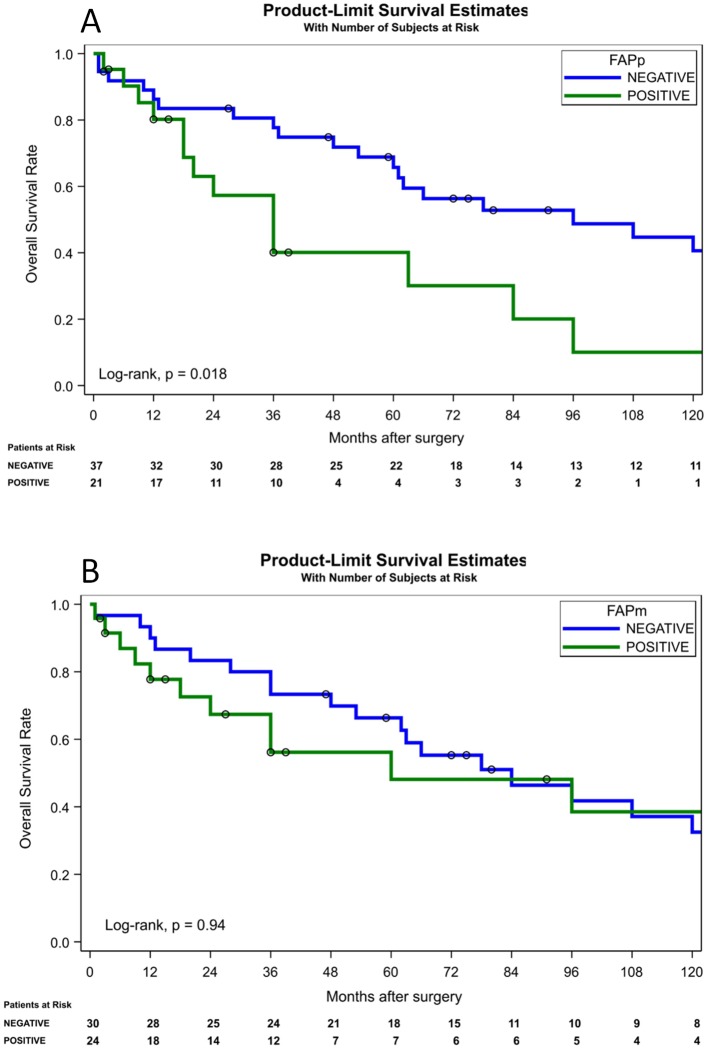
Kaplan-Meier survival curves for FAP expression in primary (A) and metastatic (B) clear cell renal cell carcinomas. 10-year overall survival showed significant differences between FAP protein positive and negative cases in primary tumors (log-rank test p<0.05).

Kaplan-Meier survival curves were performed in patients with primary and metastatic CCRCC and showed that FAP positive immunostaining in primary tumors (Fig 2A) was associated with poor survival (log-rank; p = 0.018). However, FAP positivity in metastatic lesions (Fig 2B) did not appear a prognostic factor (log-rank; p = 0.94). Cox regression multivariate analysis showed that the temporal presentation of metastases (synchronous vs. metachronous) was the only independent prognostic variable in this population of mCCRCC (p = 0.0007). However, if the dominant variable regarding the time from diagnosis to development of metastatic disease is excluded from the model, FAP positive immunostaining of the primary tumor (p = 0.0608) and initial metastatic location in lymph nodes (p = 0.139) present as determinant of prognosis in patients with mCCRCC (Table 4). Other well-admitted clinico-pathological variables, such as stage, size or grade were not predictors in this series. Tumor necrosis appeared statistically significant in univariate analysis but did not maintain in multivariate model (Table 4).

**Table 4:** Univariate and Multivariate analyses to predict overall survival of mCCRCC patients.

**UNIVARIATE ANALYSIS**					
**Variable**	**Description**	**Point Estimate**	**95% Wald Confidence Limits**		**P-value Log-rank**
Diameter	≤ 7cm vs **>** 7cm	1.18	1.63	0.44	0.61
Grouped grade	Low (G1-G2) vs High (G3-G4)	1.19	0.62	2.28	0.59
Grouped pT	Low (pT1-pT2) vs High (pT3-pT4)	1.01	0.53	1.93	0.97
Sarcomatoid	No/Yes	2.66	0.61	11.63	0.17
Necrosis	No/Yes	2.17	1.11	4.25	**0.02**
Synchronicity	Synchronous vs Metachronous	6.29	2.44	16.13	**<0.0001**
Location	Lymph node vs Epithelial tissue	1.78	1.3	0.24	0.39
	Lymph node vs soft tissue/bone	1.53	1.67	0.26	
	Epithelial vs soft tissue/bone	0.86	2.45	0.56	
	Lymph node vs rest	1.69	1.31	0.26	0.19
FAPp	Negative vs positive	2.27	1.12	4.57	**0.018**
FAPm	Negative vs positive	1.03	0.51	2.08	0.94
**MULTIVARIATE ANALYSIS**					
**Variable**	**Description**	**Point Estimate**	**95% Wald Confidence Limits**		**P-value Cox**
Synchronicity	Synchronous vs Metachronous	6.29	2.44	16.13	**0.0007**
**MULTIVARIATE ANALYSIS (Excluding Synchronicity)**					
**Variable**	**Description**	**Point Estimate**	**95% Wald Confidence Limits**		**P-value Cox**
FAPp	Negative vs positive	1.94	0.79	4.75	0.06
Location	Lymph node vs rest	1.9	0.88	4.1	0.14

Stepwise Cox proportional hazards test showed synchronicity between primary tumor and metastases was an independent prognostic variable to predict overall survival. An alternative model revealed FAPp positivity and metastatic location in lymph nodes also predicted overall survival (although it did not reach statistical significance). Statistically significant results are highlighted in bold.

## Discussion

CCRCC is a heterogeneous and complex neoplasm. Metastatic disease is present at diagnosis in around 30% of patients and another third of those with early-stage CCRCC at diagnosis will relapse and progress to metastatic disease after nephrectomy [5,16]. In the context of a metastatic disease, the importance of classic prognostic factors such as tumor diameter, stage and histological grade of the primary tumor, decreases considerably [17]. So, the identification of alterations that may influence tumor behavior and clinical outcome in primary tumors and metastases is needed to improve the management of these patients [18]. Very recently lymph node metastases have proved negative impact on the survival of mCCRCC treated with targeted therapies [19]. By contrast, the survival benefit of lymph node dissection in patients with localized disease and clinically enlarged lymph nodes has not been demonstrated and could represent overtreatment in the majority of cases. Consequently, lymph node dissection is usually performed for staging purposes [20].

Cancer development and progression rely not only in neoplastic cells themselves, but also in their interaction with CAFs in tumor microenvironment [8,9,21], among other factors. FAP is a marker of activated fibroblasts and its expression is more abundant in tumors with invasive phenotype that are more likely to metastasize [9]. The relationship between FAP expression and poor clinical outcome has been reported [11]. However, these studies have been performed in primary tumors and information about FAP expression in metastatic lesions is scarce.

In this study, we analyzed FAP expression in the stroma of both primary (FAPp) and metastatic (FAPm) lesions of the same mCCRCC patients. As reported very recently in a global series of CCRCCs [13], FAPp immunostaining significantly correlated with high stage, high grade and necrotic tumors, and implied worse 10-year overall survival. These findings are recapitulated in the population of CCRCC with metastases at diagnosis or developing metastasis during follow-up. Furthermore, we have found that there is a correlation between FAP expression in the stromal fibroblasts in primary tumor and in the metastases; however, the negative impact of FAP on survival is mainly determined by stromal fibroblasts in the primary tumor and not in the metastases.

The temporal presentation of metastases is itself an important variable influencing prognosis in cancer patients. In renal cancer, synchronous metastases are related with worse survival than metachronous ones [22]. Also concomitant lung or lymph node metastases were independent predictors in patients with CCRCC and bone metastases [23]. In our study, the temporal presentation of metastases also remained as the highest significant variable influencing the survival of mCCRCC patients. This finding has also been detected by different authors, although they describe this variable under different names, such as time from initial diagnosis in mCCRCC patients [24], time to nephrectomy to mCCRCC [25] or time from diagnosis to metastatic disease [26]. In this series, the expression of both FAPp and FAPm was positive in more than 80% of synchronous metastases, whereas it was negative in most metachronous lesions.

On the other hand, synchronous metastases occurred mainly in lymph nodes whereas metachronous were located in epithelial organs. As previously described in other cancers [11], most mCCRCCs metastasizing into the lymph nodes were FAP positive. These results suggest an early migration of cancer cells from FAP positive primary tumors to lymph nodes, and could explain, at least in part, both the shorter survival outcome of mCCRCC with lymph node metastases [19,26] and also the relationship between FAP expression and worse prognosis of mCCRCC patients.

An important number of CAFs are present closely associated with carcinoma cells in metastases as a part of the tumor microenvironment [27]. Within the lymph nodes, CAFs share similar biomarker profiles than in primary tumors [28]. In our study, FAP was also significantly expressed in nodal CAFs, suggesting that microenvironments in mCCRCC are similar in primary tumor and in lymph nodes [27,28].

FAP is a multifunctional protein that regulates tumor growth and invasiveness through mechanisms that are dependent and independent of its catalytic activity [21,29]. For example, FAP may form complexes with its homologous enzyme, dipeptidyl-peptidase (DPPIV), facilitating ECM degradation and thus promoting cancer cell invasion [21,29]. In addition, both proteins can also act as adhesion molecules, interacting with integrins and influencing the intracellular signaling of these proteins [21,30]. DPPIV is also increased in advanced CCRCC and is associated with poor survival rates in these tumors, which suggests that both FAP and DPPIV may have complementary roles in renal carcinogenesis [13,31,32].

Epithelial-to-mesenchymal transition (EMT) is a reversible process in which cancer cells loss their epithelial features and transform into mesenchymal cells, and is a crucial step in the acquisition of metastatic behavior of tumors [33]. Sarcomatoid transformation, a fact that can be observed in 8–10% of CCRCCs, represents a good example of neoplastic EMT in renal cancer [34]. Patients diagnosed with sarcomatoid CCRCC show a considerable worse prognosis due to an increased propensity for metastasis [34]. In this study, the immunostaining of FAPp and FAPm was positive in 100% of sarcomatoid CCRCCs, a finding that supports the idea that FAP and CAFs could also confer aggressive behavior to renal cancer cells by regulating EMT [35].

FAP is not found in normal adult tissues and its expression is largely associated to the stroma of primary and metastatic tumors [11,12,21]. The diagnostic and therapeutic potential of this distribution pattern is currently under study [9,36–38]. It has been demonstrated very recently that FAP-targeting fluorescent immunoliposomes can be used for intraoperative imaging and may improve the accurate and complete resection of tumors and lymph node metastases [36–37]. Besides, FAP-activated prodrugs have been tested in prostate and breast cancer with promising results [38–40]. Taking into consideration that CCRCC has only partial response to targeted therapies and that FAP is expressed in the stroma of more aggressive CCRCCs, the addition of this serine peptidase to the list of potential therapeutic targets would open new opportunities for these patients.

## Conclusions

1) FAP is expressed in CAFs from primary CCRCCs and their metastases. 2) The expression of FAP may predict early presentation of CCRCC metastases to lymphatic nodes, 3) FAP is significantly associated with tumor aggressiveness and poor outcome of patients with mCCRCC and 4) The simultaneous expression of FAP in primary and metastatic tumors merits further investigation.

## Supporting Information

S1 Fig FAPα antibody specificity was tested against human recombinat FAPα protein. The western blot analysis confirmed the detection of a unique band at the expected 85KDa molecular mass when the human recombinant FAP protein was loaded, confirming the specificity of the antibody against FAP.
(TIF)

## Abbreviations

CAF: cancer associated fibroblast
CCRCC: clear cell renal cell carcinoma
EMT: Epithelial-to-mesenchymal transition
FAP: Fibroblast Activation Protein-α
FAPm: FAP in metastases
FAPp: FAP in primary tumors
mCCRCC: metastatic clear cell renal cell carcinoma
