# Supplementary figures and images for: The Expression of Fibroblast Activation Protein in Clear Cell Renal Cell Carcinomas Is Associated with Synchronous Lymph Node Metastases
Source: PLoS One. 2016 Dec 29;11(12):e0169105. doi: 10.1371/journal.pone.0169105 (PMC5199084; doi:10.1371/journal.pone.0169105)

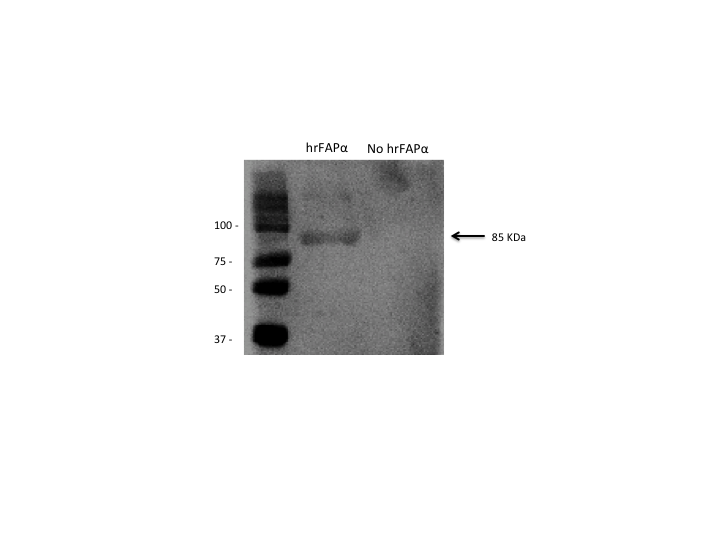

Supplement: S1 Fig — The western blot analysis confirmed the detection of a unique band at the expected 85KDa molecular mass when the human recombinant FAP protein was loaded, confirming the specificity of the antibody against FAP. (TIF) [file pone.0169105.s001.TIF]
